# Gliptin Accountability in Mucous Membrane Pemphigoid Induction in 24 Out of 313 Patients

**DOI:** 10.3389/fimmu.2018.01030

**Published:** 2018-05-24

**Authors:** Olivier Gaudin, Vannina Seta, Marina Alexandre, Gérôme Bohelay, Françoise Aucouturier, Sabine Mignot-Grootenboer, Saskia Ingen-Housz-Oro, Céline Bernardeschi, Pierre Schneider, Benoît Mellottee, Frédéric Caux, Catherine Prost-Squarcioni

**Affiliations:** ^1^Department of Dermatology, Referral Center for Autoimmune Bullous Diseases (MALIBUL), Avicenne Hospital, Assistance Publique Hôpitaux De Paris (AP-HP), Paris 13 University, Bobigny, France; ^2^Department of Dermatology, Referral Center for Autoimmune Bullous Diseases (MALIBUL), Cochin Hospital, Assistance Publique Hôpitaux De Paris (AP-HP), Université Paris Descartes, Paris, France; ^3^Department of Immunology, Referral Center for Autoimmune Bullous Diseases (MALIBUL), Saint-Louis Hospital, Assistance Publique Hôpitaux De Paris (AP-HP), Paris, France; ^4^Department of Immunology, Referral Center for Autoimmune Bullous Diseases (MALIBUL), Bichat Hospital, Assistance Publique Hôpitaux De Paris (AP-HP), Paris, France; ^5^Department of Dermatology, Referral Center for Autoimmune Bullous Diseases (MALIBUL), Henri-Mondor Hospital, Assistance Publique Hôpitaux De Paris (AP-HP), Créteil, France; ^6^Department of Dermatology, Referral Center for Autoimmune Bullous Diseases (MALIBUL), Saint-Louis Hospital, Assistance Publique Hôpitaux De Paris (AP-HP), Paris, France; ^7^Department of Histology, UFR Léonard de Vinci, Paris 13 University, Bobigny, France; ^8^Department of Pathology, Avicenne Hospital, Assistance Publique Hôpitaux De Paris (AP-HP), Paris 13 University, Bobigny, France

**Keywords:** autoimmune bullous diseases, mucous membrane pemphigoid, fibrosis, dipeptidyl peptidase IV inhibitor, gliptin, diabetes mellitus, adverse drug reaction, drug-accountability study

## Abstract

Mucous membrane pemphigoids (MMPs) and bullous pemphigoid (BP) are autoimmune bullous diseases that share physiopathological features: both can result from autoantibodies directed against BP180 or BP230 antigens. An association has been reported between BP and intake of gliptins, which are dipeptidyl peptidase-IV inhibitors used to treat type 2 diabetes mellitus. Clinical and immunological differences have been reported between gliptin-induced BPs and classical BPs: mucosal involvement, non-inflammatory lesions, and target BP180 epitopes other than the NC16A domain. Those findings accorded gliptins extrinsic accountability in triggering MMP onset. Therefore, we examined gliptin intrinsic accountability in a cohort of 313 MMP patients. To do so, we (1) identified MMP patients with gliptin-treated (challenge) diabetes; (2) selected those whose interval between starting gliptin and MMP onset was suggestive or compatible with gliptin-induced MMP; (3) compared the follow-ups of patients who did not stop (no dechallenge), stopped (dechallenge) or repeated gliptin intake (rechallenge); (4) compared the clinical and immunological characteristics of suggestive-or-compatible-challenge patients to 121 never-gliptin-treated MMP patients serving as controls; and (5) individually scored gliptin accountability as the trigger of each patient’s MMP using the World Health Organization-Uppsala Monitoring Center, Naranjo- and Begaud-scoring systems. 17 out of 24 gliptin-treated diabetic MMP patients had suggestive (≤12 weeks) or compatible challenges. Complete remission at 1 year of follow-up was more frequent in the 11 dechallenged patients. One rechallenged patient’s MMP relapsed. These 17 gliptin-treated diabetic MMP patients differed significantly from the MMP controls by more cutaneous, less buccal, and less severe involvements and no direct immunofluorescence IgA labeling of the basement membrane zone. Multiple autoantibody-target antigens/epitopes (BP180–NC16A, BP180 mid- and C-terminal parts, integrin α6β4) could be detected, but not laminin 332. Last, among the 24 gliptin-treated diabetic MMP patients, five had high (I4–I3), 12 had low (I2-I1) and 7 had I0 Begaud intrinsic accountability scores. These results strongly suggest that gliptins are probably responsible for some MMPs. Consequently, gliptins should immediately be discontinued for patients with a positive accountability score. Moreover, pharmacovigilance centers should be notified of these events.

## Introduction

Mucous membrane pemphigoids (MMPs) are rare diseases with very low annual incidences worldwide, ranging from 0.07 million inhabitants in Kuwait to 2 million inhabitants in Germany, and intermediate, with 1.25 million inhabitants, in France (1). These diseases are defined clinically. They cover a heterogeneous group of subepithelial autoimmune blistering diseases that predominantly affect the mucous membranes (2). They include the classical MMP, formerly called cicatricial pemphigoid, laminin 332 MMP, α6β4 integrin MMP, mucous membrane dominant epidermolysis bullosa acquisita (MM-EBA), and mucous membrane dominant linear IgA disease [MM-linear IgA bullous dermatosis (LABD)]. Abnormal scarring is the hallmark of MMPs: lesions heal *via* a fibrosing process leading to cicatricial lesions that can cause severe impairment of the eyes or can be life-threatening in larynx or esophagus.

Although MMP clinical characteristics differ from those of bullous pemphigoid (BP) (younger patients, mucous membrane involvement, bullous cutaneous lesions predominantly on the head-and-neck, cicatricial evolution) (3), classical MMP, and BP share physiopathological features: both result from the activity of autoantibodies directed against hemidesmosomal proteins of basal keratinocytes, BP 230 (BP230) and BP 180 (BP180) antigens, predominantly the C-terminal region and BP180–NC16A epitopes in MMP and BP, respectively (2, 4, 5).

An association between BP and the intake of several drugs (spironolactone, amiodarone, sulfasalazine, allopurinol, furosemide, etc.) has been reported, since 1970 (6–8), and most recently with gliptins, which are dipeptidyl peptidase-IV (DPPIV) inhibitors used to treat type 2 diabetes mellitus. Three gliptins are currently available in France: sitagliptin and vildagliptin, since 2007, and saxagliptin, since 2009. The first BP cases associated with gliptin intake were described in 2011. Since then, 42 cases of gliptin-associated BP have been published as case reports or in short series (9–23), 37 in two case-control studies (20, 24), and 208 identified in pharmacovigilance databases (16, 25). A study comparing 3,397 BP patients to 12,941 basocellular carcinoma controls from the Finnish nationwide registry and showing that vidagliptine increases the risk of BP has also been partially published very recently (26). Several authors have highlighted different clinical and immunological phenotypes of these gliptin-associated BPs: mucosal involvement (15), non-inflammatory lesions (18, 23), and target BP180 epitopes outside the NC16A domain (18, 23).

Because the role of gliptins in MMP had never been investigated, we examined gliptin accountability in MMP induction in 24 gliptin-treated diabetic MMP patients in our center cohort of 313 MMP patients. Our primary objective was to identify patients with a first gliptin-intake-to-MMP-onset interval “suggestive” or “compatible” with MMP induction. Then we analyzed clinical and immunological findings and outcomes of these selected patients to evaluate other accountability criteria of gliptin MMP induction and, finally, indicate prognosis.

## Materials and Methods

### Referral Center Database

This single-multisite-center retrospective study (January 2007–June 2016), approved by our local Institutional Review Board (IRB 00003835 no. 2013/39NI), was conducted using the database of our Referral Center for autoimmune bullous diseases. The following information was systematically recorded in each patient’s standardized medical chart. During their first consultation at our Center, all patients were asked about their medical history and treatments, evaluated by a multidisciplinary team that noted all cutaneous and mucous membrane lesions, clearly distinguishing MMP reversible “active” mucous membrane lesions from irreversible “cicatricial” mucous membrane lesions (27–29). Skin and/or mucous membrane biopsy findings and immunoserological results at diagnosis yielding a definite diagnosis were also recorded: direct immunofluorescence (DIF) immune-deposit pattern at the dermal–epidermal junction (linear) and Ig class(es) (IgA, IgG, IgM), ±C3 deposits; ultrastructural immune-deposit location by direct immunoelectron microscopy (IEM; when done, according to availability in each department); standard indirect immunofluorescence (IIF) on rat or monkey esophagus and 1 M NaCl-treated human or commercially available (Euroimmun, Lübeck, Germany) monkey salt-split skin (SSS), using polyvalent anti-IgG, IgA, IgM as secondary antibodies; commercially available BP180–NC16A and BP230 enzyme-linked immunosorbent assays (ELISAs) using anti-IgG secondary antibodies (MBL, Nagoya, Japan and/or Euroimmun, Lübeck, Germany); and immunoblot on amniotic membrane extracts (when done, according to availability in each department). In patients with a subepithelial autoimmune blistering disease [i.e., linear immunoglobulin (Ig) deposits along the basement membrane zone (BMZ) in DIF], diagnoses of MMP and BP were retained on clinical criteria: the first when lesions predominantly affected the mucous membranes (2) and the second in patients who fulfilled criteria of Vaillant (3). Diagnoses of LABD and EBA were retained on immunological criteria: the first on class IgA of autoantibodies and the second when autoantibodies targeted type VII collagen (by ELISA and/or immunoblot) and/or are located in anchoring-fibril zone (AFz) (by IEM). Diagnosis of MM-LABD or MM-EBA was retained in patients with predominant mucous membrane lesions and respectively a LABD or an EBA. Other MMP subgroups have been diagnosed according to the target antigen of autoantibodies (by IEM, IIF on SSS, ELISA, and/or immunoblot). In particular, the immunoblot on amniotic extract allowed the detection of antibodies to laminin 332 and α6/β4 integrin (30). Last, MMP was classified as severe or not according to Chan criteria (2, 27–29).

### Gliptin-Treated MMP Patients

First, we identified all consecutive patients with a definite MMP diagnosis, then selected those with diabetes mellitus, and, finally, included MMP patients prescribed gliptins to treat their diabetes.

### Chronology of Gliptin Intake and MMP Onset

**Figure 1:**
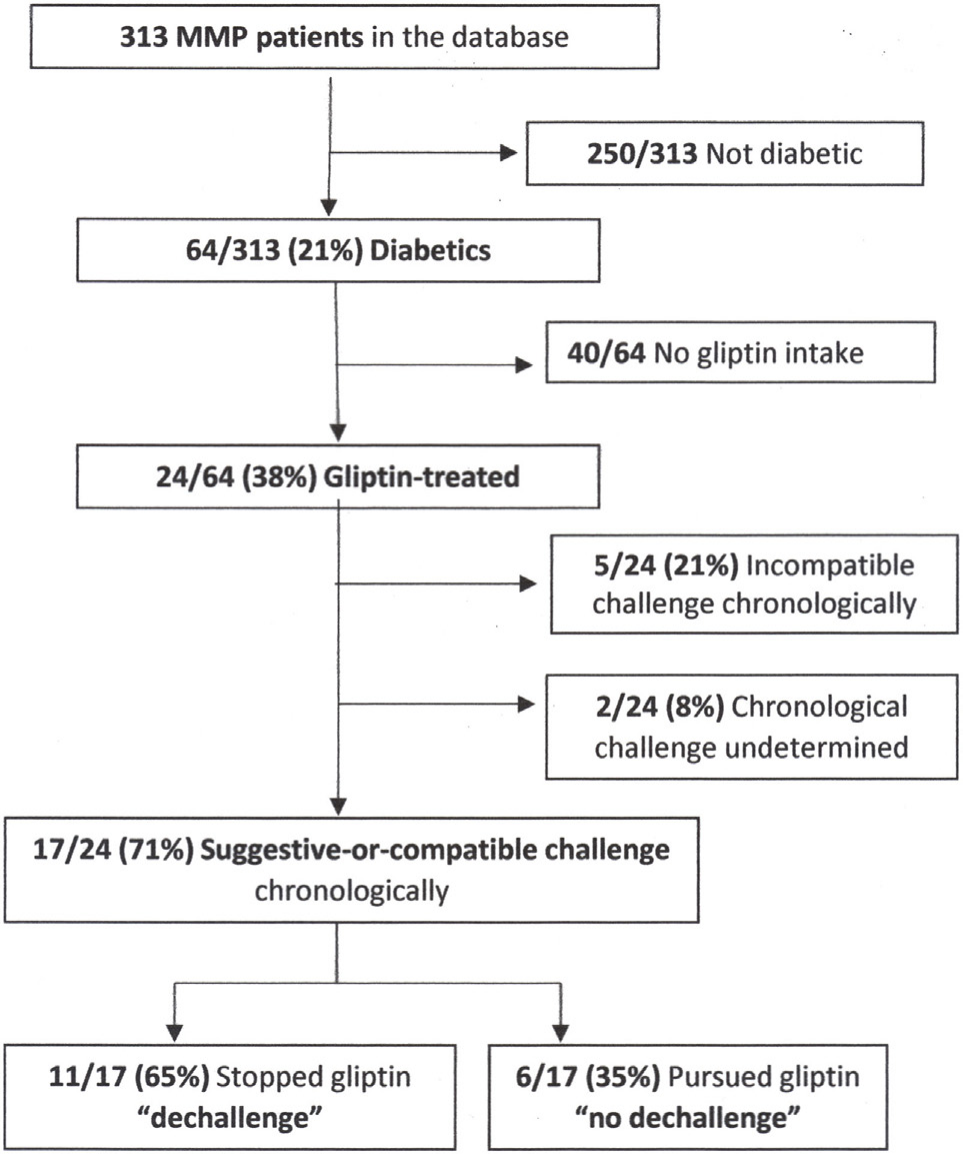
Flowchart. Abbreviation: MMP, mucous membrane pemphigoid.

Using Begaud’s updated nomenclature (**terms in bold type;** 31) to impute a potential gliptin role in triggering MMP, dates of gliptin introduction (**challenge**), discontinuation (**dechallenge**), reintroduction (if any) (**rechallenge**), and the first MMP symptoms were extracted from the Center’s database, collected from patient’s chart and/or by contacting the patient’s general practitioner. Diabetics who started taking a gliptin before MMP onset and had a first gliptin-intake-to-MMP-onset interval suggestive or compatible with MMP induction formed the **suggestive-or-compatible challenge** group (Figure 1). Those who discontinued gliptin during the first year of MMP follow-up formed the dechallenge group and those who did not comprised the **no-dechallenge** group.

### Clinical and Immunological Findings and MMP Follow-Up

Clinical and immunological characteristics at MMP diagnosis were extracted from the Center’s database. Observation end points were collected retrospectively from patients’ charts: time to first MMP complete remission (CR), CR rate at 1 year of MMP follow-up, and relapse rates at 1 year and the end of MMP follow-up (1–3 years), according to Murrell et al.’s criteria (31).

### Reference Series of MMP Patients Without Gliptin Intake

One hundred and twenty-one consecutive MMP patients who had never taken a gliptin and had at least 1 year of follow-up served as controls. Patients with MM-LABD were excluded, as were those with MM-EBA. Clinical and immunological findings at diagnosis and CR and relapse rates at 1 year of follow-up were extracted from the Center’s database and/or collected from each patient’s chart.

### Statistical Analyses

Quantitative variables, reported as mean ± SD or medians (range), were compared with Mann–Whitney *U*-tests. Qualitative parameters, expressed as numbers (%) were compared with chi^2^ or Fisher’s exact tests, as appropriate. Two-tailed *p*-values less than 0.05 were considered statistically significant. Statistical analyses were computed with R version 3.4.3 (R Foundation for statistical Computing, Vienna, Austria).

### Accountability Scoring

Accountability criteria were analyzed and scored using Begaud’s system, updated in 2011 (32, 33), Naranjo’s method (34) and the World Health Organization–Uppsala Monitoring Center’s (WHO–UMC) assessment (35).

## Results

### Gliptin-Treated MMP Patients

Among the 313 MMP patients seen in our Center between January 2007 and June 2016, 64 (20%; 39 F/25 M) were diabetics and 24 (38%) of them were treated with gliptin (Figure 1). All but four of them (patients 7, 10, 13, and 14) had >1 year of follow-up in our Center.

### Chronological Data on Gliptin Use

17 (71%) of the 24 MMP patients started taking a gliptin to treat their diabetes before MMP onset (**suggestive-or-compatible challenge** group), with the median first gliptin-intake-to-MMP-onset interval of 136 (range 4–588) weeks (Table 1). Arbitrarily, ≤12 weeks to MMP onset was considered **suggestive** of gliptin induction for patients 1–4 and >12 weeks considered **compatible** for patients 5–17. Patients 1–11 (11, 65%) of the 17 had discontinued gliptin because of uncontrolled diabetes (**dechallenge** group) and patients 12–17 (6, 35%) did not (**no-dechallenge** group). 5 (21%) of the 24 gliptin-treated MMP diabetics started taking it after MMP onset (**incompatible-challenge** group). For the remaining two patients, the date of gliptin introduction could not be determined (**undetermined-challenge** group). No other therapeutic agent was suspected in MMP onset.

**Table 1:** Clinical characteristics of the 17 MMP patients with suggestive-or-compatible gliptin challenges.

Challenge	Sex/age (years)	Weight (kg)/BMI (kg/m^2^)	1st gliptin-dose-to-MMP-onset interval (weeks)	MM sites involved						MMP severity		Initial treatment	1-year MMP follow-up	
							
Gliptin patient				Total*n*	Skin	Mouth	Genitals/anus	Eyes	NT/larynx	Mild	Severe		Relapse	CR
**Dechallenge**														
*Saxagliptin*														
1	M/79	65/24	4	4	+	+	–	+	+/+	−	+	Dap, RTX	No	Yes
*Vildagliptin*														
2	F/71	68/27	4	2	−	+	+	−	−/−	+	−	Dap	No	Yes
3	M/60	75/29	4	3	+	+	+	−	−/−	+	−	Doxy	No^a^	Unknown
4	F/71	Unknown	12	2	−	+	−	−	+/+	−	+	Dap, CyP	Yes	Yes
5	M/77	80/27	36	3	+	+	−	−	+/−	+	−	Dap	No	Yes
6	F/61	92/36	36	5	+	+	+	+	+/+	−	+	Dap, RTX	Yes^b^	No
7	M/81	80/30	144	2	+	−	−	−	+/−	+	−	Doxy	Unknown^c^	Unknown
*Sitagliptin*														
8	M/62	91/29	104	3	−	+	+	−	+/−	+	−	Dap	Yes^d^	Yes
9	F/57	Unknown	136	3	+	+	−	−	+/+	−	+	Dap, CyP	No	Yes
10	F/74	55/25	144	3	+	+	−	−	+/−	+	−	Dap	Unknown^c^	Unknown
11	M/76	Unknown	232	2	+	−	+	−	−/−	+	−	tCTC	No	Yes
**No dechallenge**														
*Vildagliptin*														
12	M/72	70/25	72	4	+	+	+	−	+/−	−	+	Dap, CyP	Yes	No
13	F/75	105/41	148	3	+	−	+	−	+/+	−	+	CyP, Doxy	Unknown^c^	Unknown
14	F/48	100/33	236	2	+	−	−	−	+/−	+	−	Dap	Unknown^c^	Unknown
15	F/71	77/nd	244	2	+	−	+	−	−/−	+	−	Unknown	No	Yes
16	F/65	100/43	588	3	+	+	−	−	+/−	+	−	Dap	No	Yes
*Sitagliptin*														
17	M/64	154/45	144	2	+	−	−	−	+/+	−	+	CyP, Doxy	Yes^a^	Unknown

*MMP, mucous membrane pemphigoid; BMI, body mass index; MM, mucous membrane; NT, nose and throat; CR, complete remission; +, positive; −, negative; Dap, dapsone; RTX, rituximab; Doxy, doxycyclin; CyP, cyclophosphamide; tCTC, topical corticosteroid*.

*^a^These patients were followed for >1 year but were not examined at 1 year, so exact status at 1 year is unknown*.

*^b^Relapse on gliptin*.

*^c^Follow-up <1 year, with no additional information about outcome*.

*^d^Relapse after rechallenge*.

### Clinical and Immunological MMP Data

**Figure 2:**
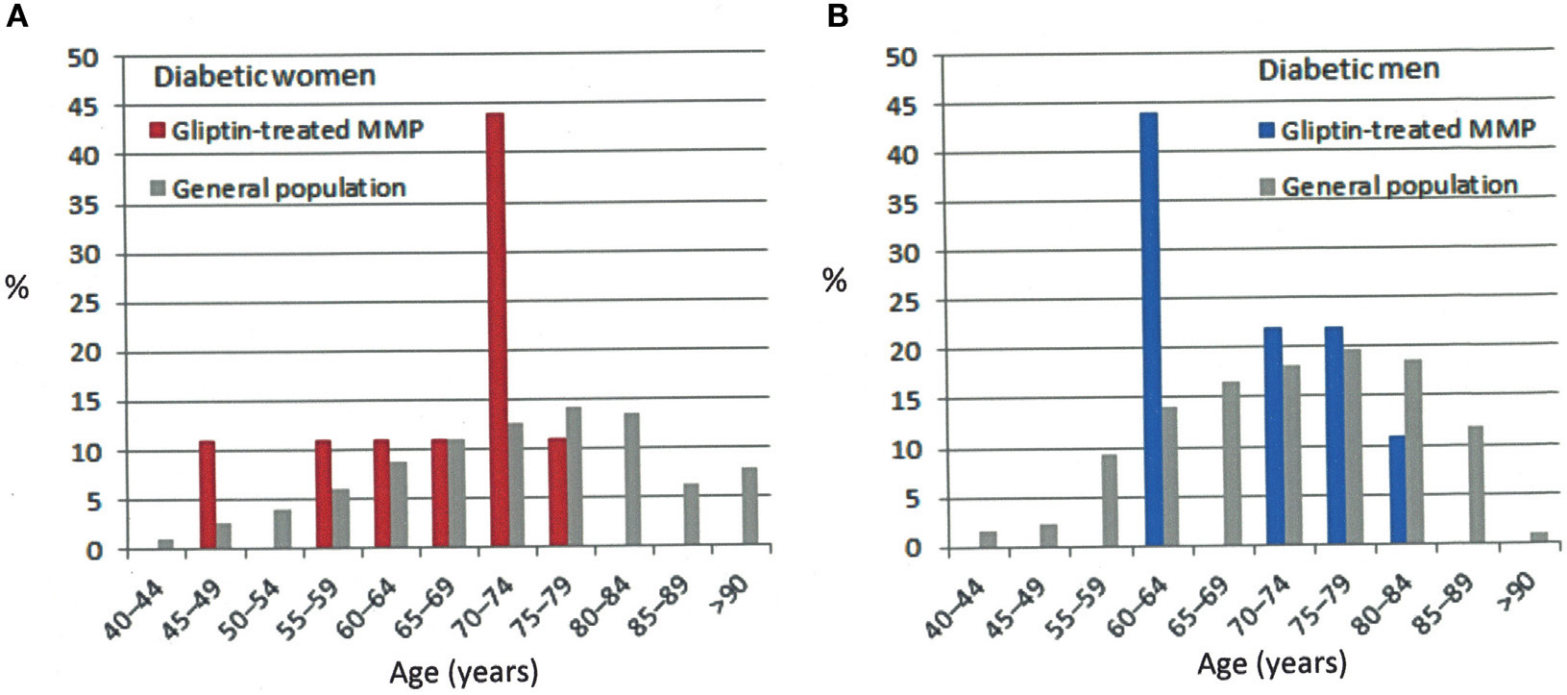
Percentage of patients by age group in the general diabetic population and our suggestive-or-compatible gliptin-induced mucous membrane pemphigoid group: **(A)** in women, **(B)** in men, diabetic patients (36).

**Figure 3:**
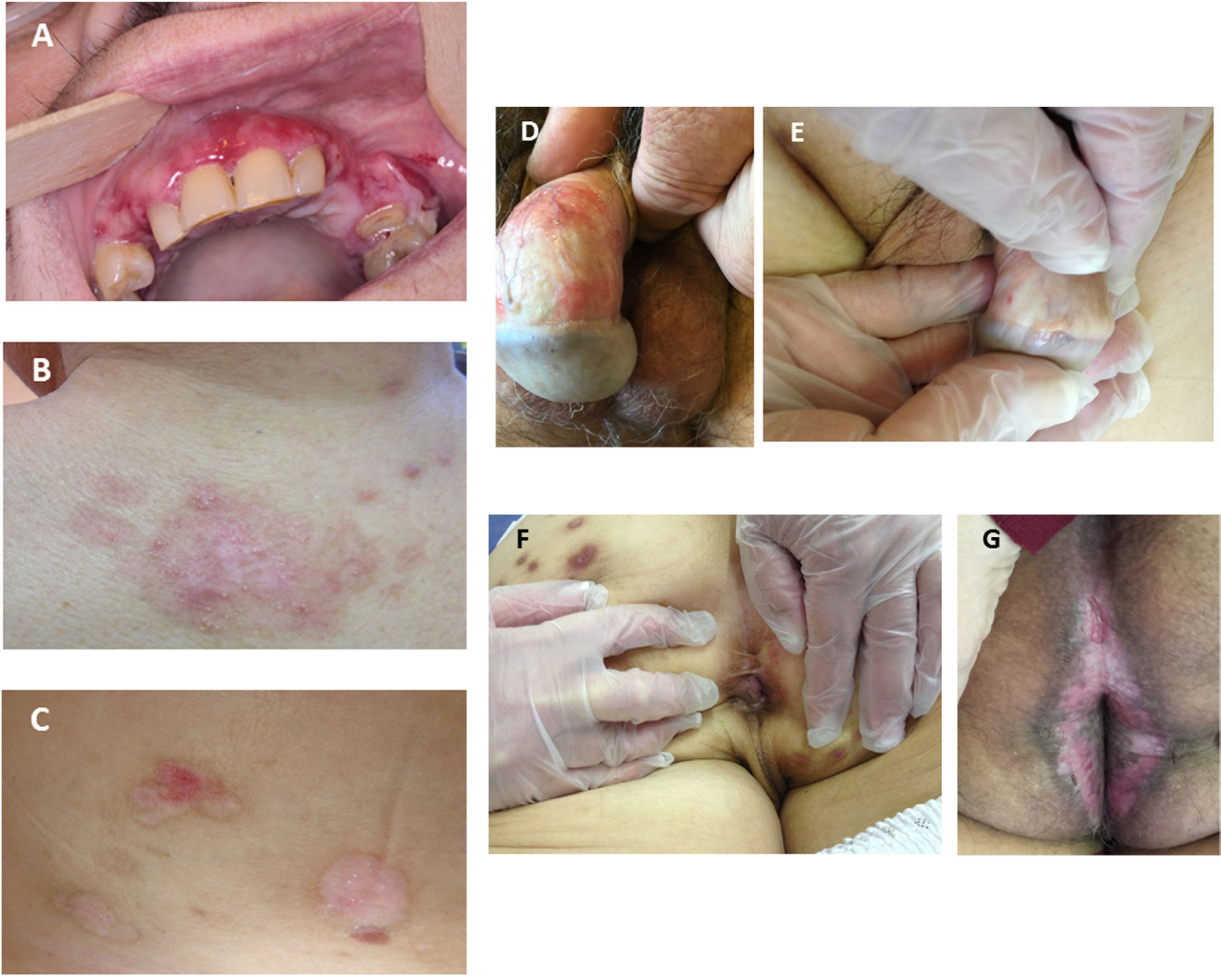
Typical patterns and locations of active and cicatricial mucous membrane pemphigoid lesions in patients 10 **(A)**, 13 **(B,E,F)**, 16 **(C)**, 8 **(D)**, and 3 **(G)**. **(A)** Active buccal mucosa lesions: erosions covered by pseudomembranes or yellowish slough, surrounded by inflammatory erythema. **(B)** Cicatricial cutaneous lesions: atrophic scars and milia on the upper back. **(C)** Active and cicatricial lesions: post-bullous erosion and atrophic scars on the breast. **(D)** Post-bullous erosions and synechiae between the prepuce and the glans penis. **(E)** Disappearance of the balanopreputial furrow. **(F)** Synechiae in perianal area and atrophic scars on the skin. **(G)** Perianal linear erosion and atrophic scars.

Among the 17 **suggestive-or-compatible-challenge** group patients (nine women; eight men), patient 1 took saxagliptin, 11 vildagliptin (patients 2–7 and 12–16), and five sitagliptin (patients 8–11, 17) (Table 1) alone or combined with metformin. At MMP diagnosis, their ages ranged from 48 to 81 (mean 69, median 71) years, 48–75 (mean 66, median 71) years for women and 60–81 (mean 71, median 74) years for men (Figure 2), their weight from 55 to 154 (mean 87, median 80) kg and their body mass indexes from 24 to 45 (mean 32, median 29). Three (18%) of them had exclusively mucous membrane involvement, and 14 had (82%) mucous membrane and cutaneous involvements. A median of three sites per patient were involved (Figure 3): skin (14, 82%), mouth (11, 65%), larynx (6, 35%), genitals and/or anus (8, 47%), and conjunctiva (2, 12%); none had esophageal involvement. Last, patients 1, 4, 6, 9, 12, 13, and 17 [seven, 41%] had severe MMP involvement, with more than three involved sites for patient 12, and laryngeal involvement in the other six associated with severe conjunctival fibrosis in patients 1 and 6. Initial treatment chosen according to the MMP severity and the patients’ comorbidities was dapsone, alone or in association with 11 patients, doxycycline with 6, cyclophosphamide with 5, and rituximab with two (Table 1).

**Figure 4:**
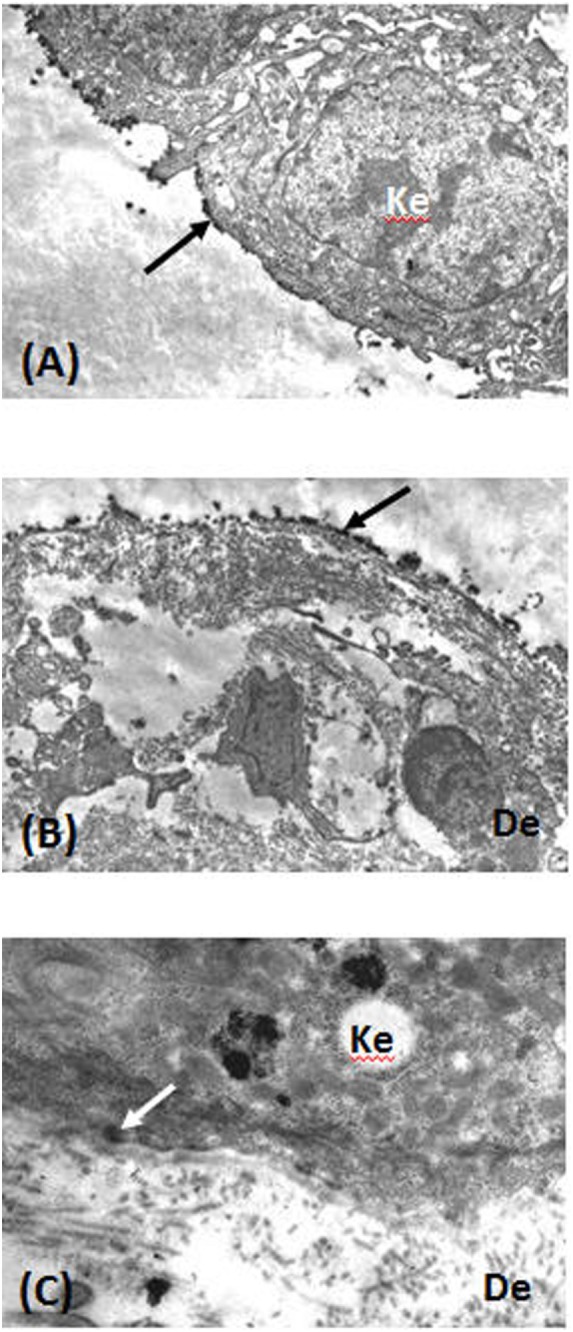
Direct immunoelectron microscopy of tissue sections from patients 10 **(A,B)** and 8 **(C)** was performed as previously described (37). **(A)** Immune deposits (arrow) on the lamina lucida (LL) cleavage roof. **(B)** Immune deposits (arrow) in the lower LL and lamina densa at the cleavage floor. **(C)** Immune deposits (arrow) in the upper LL, close to hemidesmosomes. Abbreviations: Ke, keratinocyte; De, dermis.

**Figure 5:**
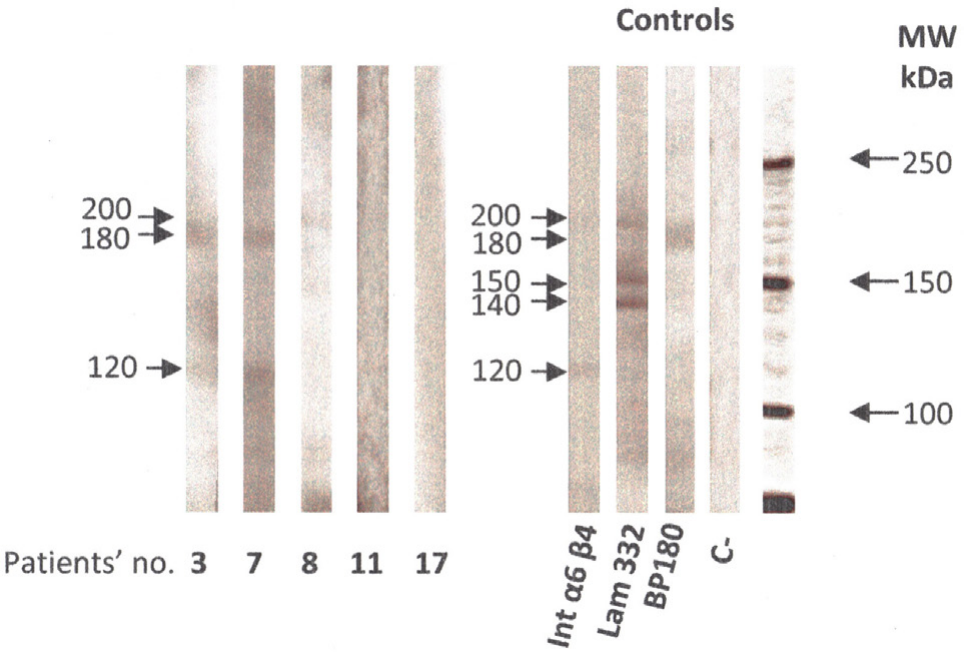
Immunoblot on amniotic membrane extract with sera from patients 3, 7, 8, 11, and 17 of the suggestive-or-compatible gliptin-induced mucous membrane pemphigoid group, was performed as previously described (30). Positive controls were α6 β4 integrin, laminin 332, and BP 180 antigen; C− was a negative control with normal human serum. Abbreviation: MW, molecular mass.

Direct immunofluorescence microscopy of tissue samples from 14 (82%) of the 17 of suggestive-or-compatible-challenge group patients had linear IgG deposits and 15 (88%) linear C3 deposits along the BMZ; none had IgA deposits (Table 2). IEM (Figure 4) of nine patients’ biopsies were positive for eight (89%): immune deposits were located on the lamina densa with/without the lamina lucida (LL) in seven (78%) of them and exclusively on the upper LL in the last one (11%); none had deposits under the lamina densa in the AFz. Autoantibodies directed against BMZ antigens were detected in 8 (47%) of the 17 patients whose sera were tested by IIF on rat or monkey esophagus. IIF on SSS was positive for 4 out of 15 patients and autoantibodies always labeled the cleavage roof; none labeled the cleavage floor. ELISA BP180 and BP230 were positive for 7 (47%) and 3 (20%) of the 15 patients tested, respectively. Immunoblots on amniotic membrane extract (Figure 5) of sera from seven of the nine ELISA BP180–NC16A-negative patients were negative for three patients and positive for four, detecting: a 200-kDa band consistent with the β4 chain of α6β4 integrin (patient 8), whose immune deposits were located on hemidesmosomes by direct IEM; a 180-kDa band (patient 3); a 120-kDa band (patient 11); and 180- and 120-kDa bands (patient 7).

**Table 2:** Immunological findings of the 17 gliptin-treated MMP patients with suggestive-or-compatible challenges.

Patient	Immune deposits on						IIF anti-BMZ IgG (esophagus)		IIF on SSS		ELISA (nl <9 AU)		Blot^a^ (kDa)
					
	DIF BMZ			Direct IEM			Rat	Monkey	Roof	Floor	BP230	BP180	
									
	IgA	IgG	C3	LD ± LL	Upper LL ± HD	AFz							
1	−	+	+	nd	nd	nd	−	−	−	−	<9	189	nd
2	−	+	+	−	−	−	−	−	−	−	1	0	nd
3	−	+	+	+	−	−	−	1/100	−	−	7	10	180
4	−	+	+	nd	nd	nd	1,280	−	+	−	61	149	nd
5	−	+	+	+	−	−	−	nd	−	−	0	1	−
6	−	−	+	+	−	−	200	nd	+	−	2	136	nd
7	−	+	+	+	−	−	−	50	−	−	0	1	180, 120
8	−	+	−	−	+	−	−	−	−	−	nd	nd	200
9	−	+	+	nd	nd	nd	−	20	+	−	2	1	−
10	−	+	+	+	−	−	200	nd	nd	nd	10	68	nd
11	−	+	+	nd	nd	nd	640	nd	+	−	−	−	120
12	−	+	+	nd	nd	nd	100	100	−	−	6	24	nd
13	−	−	+	nd	nd	nd	−	−	nd	nd	nd	nd	nd
14	−	−	+	nd	nd	nd	−	−	−	−	2	109	nd
15	−	+	+	+	−	−	−	−	−	−	8	2	nd
16	−	+	−	+	−	−	−	−	−	−	12	62	nd
17	−	+	+	nd	nd	nd	−	−	−	−	0	1	−

*MMP, mucous membrane pemphigoid; DIF, direct immunofluorescence; BMZ, basement membrane zone; Ig, immunoglobulin; IEM, immunoelectron microscopy; LD, lamina densa; LL, lamina lucida; ±, with or without; HD, hemidesmosome; AFz, anchoring-fibril zone; IIF, indirect immunofluorescence; SSS, salt-split skin; ELISA, enzyme-linked immunosorbent assay; nl, normal; AU, arbitrary unit; nd: not determined; +, positive; −, negative*.

*^a^Immunoblot on amniotic membrane extract*.

### Follow-Up of the Suggestive-or-Compatible-Challenge Group

Overall, median follow-up was 40 (range 0–164) weeks and median time to CR 8 (range 0–36) weeks. After the first year of follow-up, CR and relapse rates were 82 and 31%, respectively (Table 3). No patient died.

**Table 3:** Evolutive characteristics of the suggestive-or-compatible challenge MMP patients who discontinued gliptin (dechallenge) vs. those who continued it (no dechallenge).^a^

Characteristic	Overall		Dechallenge		No dechallenge	
			
	*n* = 17	Missing	*n* = 11	Missing	*n* = 6	Missing
**Weeks, median (range)**						
Gliptin-onset-to-MMP-diagnosis interval	136 (4–588)	0	36 (4–232)	0	136 (4–588)	0
Time to first complete remission	8 (0–36)	7	8 (2–16)	3	18 (0–36)	4
Length of follow-up	40 (0–164)	0	32 (0–104)	0	78 (4–164)	0
Initial treatment	17	1	11	0	5	1
Dapsone	11		8 (73%)		3 (50%)	
Doxycyclin	6		4 (36%)		2 (33%)	
Cyclophosphamide	5		2 (18%)		3 (50%)	
Rituximab	2		2 (18%)		0	

**At the first year of follow-up, patients, *n* (%)**						
Relapses/flares						
Yes	4 (31%)	4	2^b^ (22%)	2	2 (50%)	2
No	9 (69%)		7^c^ (78%)		2 (50%)	
Complete remission						
Yes	9 (82%)	6	7 (88%)	3	2 (66%)	3
No	2 (18%)		1 (13%)		1 (33%)	
Deaths						
Yes	0	6	0	3	0	3
No	11 (100%)		(100%)		3 (100%)	

*MMP, mucous membrane pemphigoid*.

*^a^The dechallenge group stopped gliptin intake during the first year of follow-up; the no-dechallenge group was still taking gliptin at 1 year of follow-up*.

*^b^One relapsed before and one after stopping gliptin*.

*^c^One relapsed after 1 year of follow-up and a rechallenge*.

When **dechallenge** (patients 1–11) and **no-dechallenge** (patients 12–17) groups were compared, respectively, the CR rate was higher for the former than latter (88 vs. 66%). Conversely the follow-up durations [32 (0–104) vs. 78 (4–164) weeks], times to first (CR) [8 (2–16) vs. 18 (0–36) weeks] and relapse rates of 22 vs. 50% were lower in **dechallenge** group than in **no-dechallenge** one. It is worth noting that **dechallenge** group patient 6 relapsed before gliptin withdrawal and patient 4 after it, and patient 8 relapsed 17 months after MMP diagnosis and 1 month after gliptin **rechallenge**.

Last, for the **dechallenge** group, MMP evolution was **suggestive** of gliptin imputability for six (1, 2, 5, 8, 9, and 11) of the seven patients who obtained CR, **non-suggestive** for the patient who did not, **inconclusive** for patient 4 in CR at 1 year of follow-up but who relapsed, and for patients 3, 7, and 10, with <1 year of follow-up, and patient 3 not seen at 1 year. For the **no-dechallenge** group, at 1 year of follow-up, MMP evolution was **non-suggestive** for patients 15 and 16 in CR, **suggestive** for patient 12 who had not achieved CR and **inconclusive** for remaining patients 13, 14, because of too short follow-up, and 17, who was not seen at 1 year.

### Control MMP Patients Without Gliptin Use

Our controls were 121 patients with MMP (excluding MM-EBA and MM-LABD) seen consecutively in our Center, who had never taken a gliptin and were followed for at least 1 year (Table 4). At MMP diagnosis (baseline), their ages ranged from 38 to 96 years, weights from 44 to 114 kg, and body mass indexes from 18 to 40. Half of them had only mucous membrane involvement and the other half had cutaneous and mucous membrane involvements, and 78 had severe MMP. Controls had a median of 2 (1–5) involved sites: 50% skin, 89% mouth, 30% larynx, 31% genitals/anus, 25% conjunctiva, and 31% esophagus. Immunologically, by DIF microscopy, 74% controls had linear IgG deposits along the BMZ, 71% had linear C3 deposits, and 26% had linear IgA deposits which were neither isolated nor predominate over IgG. Immune deposits were located on the lamina densa with/without the LL in 60% of them and on the upper LL in 13%. IIF microscopy of their biopsies identified circulating autoantibodies labeling the cleavage roof of SSS in 21%, the cleavage floor 2%, both sides in 3%, and no labeling of 74%. Control MMP patients whose sera labeled the cleavage floor in IIF on SSS had deposits on the lamina densa, but not the AFz by IEM, thereby excluding EBA and consistent with a laminin 332 MMP diagnosis. ELISA detected circulating anti-BP180 autoantibodies in 43 (51%) and anti-BP230 in 10 (13%) control sera. At 1 year of follow-up, 56% of the controls were in CR, 23% suffered relapses/flares, and 2% had died.

**Table 4:** Characteristics and comparisons of suggestive-or-compatible challenge vs. never-gliptin-treated control MMP groups.

Characteristic	Suggestive-or-compatible challenge		Controls		*p*
			
	*n* = 17	Missing	*n* = 121	Missing	
Age, mean/median (range), years	69/71 (48–81)		66.4/66 (38–96)		0.46
Weight, mean/median (range), kg	87/80 (55–154)		72.6/73 (44–114)		0.02
BMI, mean/median (range), kg/m^2^	32/29 (24–45)		26.1/25 (18–40)		0.01
Female/male, *n* (%)*;* sex ratio	9 (53%)/8 (47%); 1.1	0	69 (57%)/52 (43%); 1.3	0	
Involved sites, mean/median (range), *n*	2.8/3 (2–5)		2.2/2 (1–5)		
MMP involvement, *n* (%)		0		2	
Isolated MM	3 (18%)		59 (50%)		
MM and cutaneous	14 (82%)		60 (50%)		
DIF, yes/no, *n* (%)					
IgA deposits	0	0	26 (22%)/94 (78%)	1	0.04
IgG deposits	14 (82%)/3 (18%)	0	89 (74%)/31 (26%)	1	
C3 deposits	14 (82%)/3 (18%)	0	85 (71%)/35 (29%)	1	
Direct IEM, *n* (%)					
LD ± LL	7 (87%)	9	62 (60%)	18	
Upper LL ± HD	1 (13%)		13 (13%)		
IIF on SSS, *n* (%)					
Roof	4 (27%)	2	22 (21%)	18	
Floor	0		2 (2%)		
Mixed	0		3 (3%)		
Negative	11 (73%)		76 (74%)		
ELISA, positive/negative, *n* (%)					
BP230	3 (20%)/13 (80%)	2	10 (13%)/68 (87%)	43	
BP180	7 (47%)/8 (53%)	2	43 (51%)/41 (49%)	37	
Involvement, yes/no, *n* (%)					
Cutaneous	14 (82%)/3 (18%)		60 (50%)/59 (50%)		0.02
Buccal	11 (65%)/6 (35%)	0	101 (89%)/12 (11%)	8	0.01
Laryngeal	6 (35%)/11 (65%)	0	34 (30%)/79 (70%)	8	
Genital and/or anal	8 (47%)/9 (53%)	0	35 (31%)/78 (69%)	8	
Conjunctival	2 (12%)/15 (88%)	0	28 (25%)/85 (75%)	8	0.36
Esophageal	0	0	6 (5%)/107 (95%)	8	
Severe	7 (41%)/10 (59%)	0	78 (67%)/39 (33%)	8	0.05
At 1 year of follow-up, yes/no, *n* (%)					
Complete remission	9 (82%)/2 (18%)	6	68 (56%)/53 (44%)	0	0.12
Relapses/flares	4 (33%)/8 (67%)	5	28 (23%)/93 (77%)	0	
Deaths	0/17 (100%)	0	2 (2%)/119 (98%)	0	

*MMP, mucous membrane pemphigoid; BMI, body mass index; MM, mucous membrane; DIF, direct immunofluorescence; Ig, immunoglobulin; IEM, immunoelectron microscopy; LD, lamina densa; LL, lamina lucida; ±, with or without; HD, hemidesmosome; IIF, indirect immunofluorescence; SSS, salt-split skin; ELISA, enzyme-linked immunosorbent assay*.

Comparing the **suggestive-or-compatible-challenge** group’s characteristics to those of the MMP controls, most were comparable, especially the relapse and CR rates at 1 year of follow-up. The **suggestive-or-compatible-challenge** patients were older than controls but not significantly so. However, **suggestive-or-compatible-challenge** group differed significantly from controls by weighing more, having higher body mass indexes, with more frequent cutaneous involvement, less frequent buccal, and severe involvements, and no IgA deposits.

### Accountability Scoring

Using Begaud’s scoring system, **extrinsic accountability** was rated B2 (sparse and/or unreliable publications) for all our gliptin-treated MMP patients, in analogy with reported gliptin-induced BP, a similar autoimmune bullous disease (Table 5). For the **suggestive-or-compatible-challenge** group, the **chronological accountability** criterion was scored C3 (**likely**): for patients 1 and 2 because of their **suggestive** times to MMP onset and **suggestive** outcomes after gliptin **dechallenge**, and patient 8 because of the **compatible** time to MMP onset, positive **rechallenge**, and **suggestive** outcome; C2 (**plausible**): for patients 3 and 4 with **suggestive** times to MMP onset and **inconclusive** outcomes, and patients 5, 9, 11, 12, and 17 with **compatible** times to MMP onset and **suggestive** outcomes; and C1 (**doubtful**) for the seven others because of **compatible** times to MMP onset but **inconclusive** or **non-suggestive** outcomes.

**Table 5:** Gliptin accountability scores for suggestive-or-compatible MMP-induction patients.

Challenge	1st gliptin dose to MMP onset (wk)	Challenge^a^	Dechallenge		Rechallenge R0/R^**+**^/R^–^	Begaud’s accountability scores^b^			Naranjo’s score^c^	
						
gliptin patient			Yes/no	Outcome		C1–C3	S1–S3	I1–16	0–13	ADR
**Dechallenge**										
*Saxagliptin*										
1	4	Suggestive	Yes	Suggestive	R0	C3	S1	I4	3	Possible
*Vildagliptin*										
2	4	Suggestive	Yes	Suggestive	R0	C3	S1	I4	3	Possible
3	4	Suggestive	Yes	Inconclusive	R0	C2	S1	I2	2	Possible
4	12	Suggestive	Yes	Inconclusive	R0	C2	S1	I2	2	Possible
5	36	Compatible	Yes	Suggestive	R0	C2	S1	I2	3	Possible
6	36	Compatible	Yes	Not suggestive	R0	C1	S1	I1	2	Possible
7	144	Compatible	Yes	Inconclusive	R0	C1	S2	I2	2	Possible
*Sitagliptin*										
8	104	Compatible	Yes	Suggestive	R^+^	C3	S1	I4	6	Probable
9	136	Compatible	Yes	Suggestive	R0	C2	S1	I2	3	Possible
10	144	Compatible	Yes	Inconclusive	R0	C1	S1	I1	2	Possible
11	232	Compatible	Yes	Suggestive	R0	C2	S2	I3	3	Possible

**No dechallenge**									
*Vildagliptin*										
12	72	Compatible	No	Suggestive	R0	C2	S1	I2	2	Possible
13	148	Compatible	No	Inconclusive	R0	C1	S1	I1	2	Possible
14	236	Compatible	No	Inconclusive	R0	C1	S2	I2	2	Possible
15	244	Compatible	No	Not suggestive	R0	C1	S2	I2	2	Possible
16	588	Compatible	No	Not suggestive	R0	C1	S1	I1	2	Possible
*Sitagliptin*										
17	144	Compatible	No	Inconclusive	R0	C2	S2	I3	2	Possible

*MMP, mucous membrane pemphigoid; wk, week; R0, no rechallenge; R^+^, positive rechallenge; R^–^, negative rechallenge; ADR, adverse drug reaction*.

*^a^Suggestive, time to onset ≤12 weeks; compatible, time to onset >12 weeks*.

*^b^Chronological score: C1, doubtful; C2, plausible; C3, likely. Symptomatological scoring: S1, doubtful; S2, plausible; S3, likely. Intrinsic accountability scoring [combining chronological (C) and symptomatological (S) scores]: I1 (C1S1), I2 (C1S2 or C2S1), I3 (C2S2), I4 (C1S3 or C3S1), I5 (C2S3 or C3S2), and I6 (C3S3) (32)*.

*^c^Narango’s score: >9, definite ADR; 5–8, probable ADR; 1–4, possible ADR; 0, doubtful ADR (34)*.

The **symptomatological accountability** criterion was scored: S2 (**evocative**) for five patients whose clinical (cutaneous lesions, no buccal disease, no severe involvement) and immunological features (no IgA deposits), which differed significantly from controls; and S1 (**not evocative**) for the 12 other patients. No specific laboratory test can prove the link between gliptin intake and MMP.

Finally, the **intrinsic accountability** (combining C and S scores) was rated I4 for three patients, I3 for two patients, I2 for eight, I1 for four, and I0 for the seven patients with chronologically incompatible or undetermined challenge.

Naranjo’s accountability score assigns points according to the following information: (1) previous reports described a similar adverse drug reaction (ADR) (gliptin-induced BP) (1 point); (2) MMP appeared after gliptin intake (2 points); (3) MMP regressed faster after gliptin withdrawal (1 point) (patients 1–4, 8, 9, 11); (4) the adverse event appeared when the drug was readministered (2 points) (patient 8); (5) MMP that could have been idiopathic (−1 point); (6) no placebo was given (0 points); (7) the drug concentration in blood was not tested (0 points); (8) no dose-related reaction was sought (0 points); (9) a patient had the same reaction as when previously exposed (1 point) (patient 8); (10) no objective test assessed the adverse event (0 points). The Naranjo’s score was 6 for patient 8, meaning a **probable** ADR, but 3 for 5 patients and 2 for 11 patients meaning **possible** ADRs.

According to WHO–UMC accountability criteria, gliptin was **probably** responsible for triggering MMP for all patients of **the suggestive-or-compatible-challenge** group, a reasonable time relationship between drug intake and first MMP manifestations; MMP regressed after gliptin withdrawal and relapsed after readministration; and because MMP could have been spontaneous.

## Discussion

Our novel study on gliptin accountability in MMP induction was undertaken because of their extrinsic accountability, based on the following reports: MMP and BP have clinical and immunological similarities (4), a demonstrated significant association between gliptin intake and BP onset in diabetic patients (16, 24, 25) and some gliptin-associated BPs have atypical clinical and immunological phenotypes (15, 18, 23, 25).

Mucous membrane pemphigoid and BP are subepithelial AIBDs, characterized by linear immune deposits along the BMZ, but have different clinical features. MMP is clinically defined by the predominance of mucous membrane lesions over skin lesions (2) and healing of its lesions leads to characteristic cicatricial scarring. BP, on the other hand, is typified by the absence of mucous membrane lesions, absence of predominant head-and-neck involvement, and absence of scars, and older age at onset (>70 years) (3).

Mucous membrane pemphigoid and BP share two autoantibody-target antigens, BP230 and BP180, but the dominant BP180 epitopes differ (4). The majority of MMP patients’ sera react with the C-terminal domain of BP180, located in the lamina densa, combined or not with reactivity against the NC16A epitope, which is the membrane-proximal non-collagenous region of the BP180 ectodomain in upper LL (5, 38, 39). Conversely, 80–90% of BP patients have IgG autoantibodies directed against the NC16A domain (40–42). Moreover, many authors reported that variable percentages (10–50%, depending on the study) of BP autoantibodies targeted BP180 regions outside the NC16A domain (23, 43–49). Notably, that reactivity with extracellular epitopes of the BP180 C-terminal domain appeared suggestive of atypical BP, i.e., with skin and MM involvements (44, 46) or lesions limited to the lower legs and scarring of the toenail beds (49).

Other target antigens associated with the clinical MMP phenotype have been characterized molecularly: laminin 332, both α6β4 integrin subunits, and type VII collagen (4), respectively defining laminin 332 MMP, α6β4 integrin MMP, and MM-EBA. MMP also includes MM-LABD, with predominant IgA immune deposits along the BMZ.

Potential drug induction of autoimmune bullous diseases has been known for decades. Although autoimmune bullous diseases are rare diseases with low annual incidences, among which BP is the least rare, associations have been published between drug intake and many BP (6–8) and LABD cases (50) but only a few MMPs (51). Since 2011, an increasing number of reports have suggested that gliptins trigger BP (9–15, 17–23). Last, very recently, four comparative case–non-case studies demonstrated a significant association between gliptin intake and BP onset in diabetic patients (16, 24–26). To the best of our knowledge, possible gliptin-induced MMP has not been described to date.

Gliptins are DPPIV inhibitors used to treat diabetes, since 2007 in France (52, 53). They inhibit incretin degradation, which improves β-cell function in diabetics (54) by increasing insulin-secretory tone (55). Their HbA1c-lowering ability is less than that of hypoglycemic sulfonamides and glucagon-like peptide-1 inhibitor but they carry a lower risk of hypoglycemia (56).

Dipeptidyl peptidase-IV is not specific to insulinotropic hormones. It is abundantly distributed, notably in the skin, on the surface of keratinocytes, sebocytes, fibroblasts, and T cells. DPPIV is involved in the regulation of DNA synthesis and cytokine production by those cells, for example, CCL11/eotaxin (57) and transforming growth factor-β1 (TGF-β1) (58–60). DPPIV is also a cell-surface plasminogen receptor that activates plasminogen conversion leading to more plasmin (61), which is a major serine protease known to cleave the 120-kDa ectodomain of BP180, thereby generating LABD-97 antigen (62). Role of eotaxin and plasminogen–plasmin system is well known in BP pathogenesis (63, 64) and that of TGF-β1 in MMP is suspected (65). How gliptins induce BP or MMP by acting as DPPIV inhibitors on eotaxin, TGF-β1, and/or plasminogen/plasmin system remains to be elucidated.

Between 2011 and 2017, 14 case reports or small series reported 42 patients who developed BP while taking gliptins for their diabetes (Table S1 in Supplementary Material). The authors of those original articles individually scored gliptin accountability for each patient with the WHO–UMC system for 17 of them (10, 15, 20) and Naranjo’s score for 6 (19), Karch-Lasagna system for 1 (16), and accountability was assigned *a posteriori* for the remaining 18 (9, 11–14, 17, 18, 21, 22) (See Table S1 in Supplementary Material). 17 BPs were **probable** gliptin-induced ADRs, 23 were **possible** ADRs, and 2 BPs were most likely not gliptin-induced because of long interval (>48 months) between gliptin intake and BP onset.

19 (45%) out of those 40 **probable**-or-**possible** gliptin-induced BPs appeared to be associated with vildagliptin, 10 (24%) with sitagliptin, 8 (19%) with linagliptin, and 5 (12%) with another gliptin (See Table S2 in Supplementary Material). Their overall characteristics were as follows: 19 women and 21 men (F/M sex ratio 0.90), median age 76 (59–93) years, and median gliptin-intake-to-BP-onset interval 32 (4–192) weeks. Gliptin **dechallenge** for 33 had favorable outcomes for 26 and were, therefore, considered **suggestive** of gliptin imputability. One patient died 14 days after starting corticosteroids. Information on evolution after **dechallenge** was not available for five.

The first two comparative case–non-case studies were published in 2016, after analysis of pharmacovigilance databases (16, 25). Comparing French patients with BP ADRs to those with non-BP ADRs, Béné et al. showed that the former were associated more significantly and frequently with gliptin exposure (odds ratio 67.5; 95% CI 47.1–96.9) and vildagliptin carried a higher risk than other gliptins. The individual gliptin accountability for those 42 BPs was rated as **probable** for 10 and**possible** for 31, and not reported for one (Table S2 in Supplementary Material). In the European pharmacovigilance database, Garcia et al. identified 166 BPs reportedly induced by gliptin exposure. Using proportional reporting ratios, they found that BP was relatively more frequently associated with gliptins than with other drugs, again with vildagliptin being most strongly associated. Unfortunately, detailed clinical features were not reported.

Recently, a third well-designed case–non-case study was published (24). Comparing 61 diabetic BP patients to 122 age- and sex-matched diabetic controls, those authors demonstrated a significant association between gliptin use and BP onset in univariate analysis and after adjustment: 28 (46%) of the 61 diabetic BP patients took gliptins vs. 18% of the diabetic controls (odds ratio 2.64, 95% CI 1.19–5.85; *p* = 0.02 in multivariable analysis). Stratified analyses showed a stronger association for men and patients ≥80 years old. Once again, vildagliptin had a stronger association but the study was underpowered to detect differences among the other gliptins.

Very recently, a study comparing 3,397 BP patients with 12,941 basocellular carcinoma controls from the Finnish nationwide registry has shown that vidagliptine and BP are significantly associated with an adjusted odds ratio of 10.4 (4.56; 23.80) (26). The Gliptin-onset-to-BP-diagnosis interval was of 449 days. Clinical and immunological data were not available in this study.

Another case-control study (20) comparing gliptin-treated diabetic patients with BP to diabetic patients without skin diseases found more frequent gliptin use among BP diabetics [9/23 (39.1%) vs. 57/170 (33.5%)], but not significantly so.

Last, some reportedly gliptin-associated BPs had atypical clinical and/or immunological phenotypes, raising doubts about the BP diagnosis. Izumi et al. (23) described seven gliptin-associated BPs and showed they differed significantly from conventional BP by the absences of inflammatory lesions and circulating autoantibodies targeting the BP180–NC16A epitope and the presence of autoantibodies targeting the mid-portion of BP180 (120-kDa ectodomain and LABD-97). However, those patients had no autoantibodies targeting the BP180 C-terminal domain, which could have suggested an MMP diagnosis. Sakai et al. (18) also had a patient with similar gliptin-associated BP. Mendoça et al. (15) reported a patient with mucous membrane involvement at diagnosis, including arytenoid edema with several ulcerated lesions covered with fibrin, raising the question of gliptin-induced MMP rather than BP.

Finally, seven cases reported in the French pharmacovigilance database as BP ADRs to gliptin were excluded from Béné et al.’s study (25) because they did not meet Kershenovich BP, and Vaillant BP criteria, suggesting that they might really have been MMP.

This retrospective, monocenter study on a historical cohort was limited by MMP rarity. However, because our Center recruits patients with autoimmune bullous diseases, our findings should provide a fairly accurate appreciation of this population. Its retrospective design often means that data collection was incomplete and, indeed, some patients immunological test results are missing. In addition, we did not compare diabetic MMP cases to diabetic controls and epitope mapping of autoantibody-targeted antigens was not done.

The potential of gliptins to induce MMP was not investigated previously. We identified 24 gliptin-treated diabetic MMP patients, representing 38% of all MMP diabetic patients, a rate similar to that of gliptin-triggered BP in diabetics (24). We evaluated chronological gliptin accountability in MMP induction case by case: it was **incompatible**, excluding gliptin’s role, for 5 patients but **suggestive or compatible** for 17.

Vildagliptin was the most frequently incriminated gliptin for our 17 MMP diabetic patients, as for BP in the literature (Table S2 in Supplementary Material), but the highest intrinsic accountability scores were equally distributed among gliptins. It is worth noting that sitagliptin (and not vildagliptin) is the most prescribed in France, and the rest of Europe (66), suggesting that vildagliptin has a greater capacity to induce autoimmune bullous diseases.

The female/male ratio of these 17 MMP diabetics was higher than that of the general diabetic population and BP diabetics (1.1 vs. 0.7 vs. 0.65–0.83, respectively) but similar to that of our MMP controls (Table 4). MMP diabetics were younger than the general diabetic population (36) and BP diabetics tended to be older than our MMP controls. Median time to MMP onset was longer, with a wider range, than for gliptin-induced BP, which can be explained by the insidious evolution of mucous membrane lesions in MMP. The arbitrarily chosen time to distinguish **suggestive** (4 patients) from **compatible** (13 patients) chronology was ≤12 weeks; the 13 patients with **compatible** chronologies had intervals exceeding 36 weeks.

At MMP diagnosis, most of our 17 MMP diabetics did not have severe involvement. They differed significantly from MMP controls by their higher weights and their body mass indexes. Indeed, overweight and obesity is known to be a risk factor for type 2 diabetes. This difference was, therefore, expected from our population of type 2 diabetic MMP patients. They also had more frequent cutaneous involvement, less buccal involvement, and absence of DIF-detectable IgA deposits along the BMZ. The MMP outcomes of these 17 patients, during and at the end of the first year of follow-up, were the same as that for MMP controls. Indeed, gliptin-associated MMPs responded well to usual treatments after gliptin withdrawal. Intriguingly, the endocrinologists had discontinued gliptins for all the 11 patients because of insufficient diabetes control. Gliptin-triggering of MMP had never been suspected by dermatologists treating MMP patients.

Our immunological study results suggested that *in vivo-*fixed and circulating autoantibodies targeted multiple BMZ antigens/epitopes. IEM showed immune deposits in the lamina densa with/without the LL as in “classical MMP” in seven patients, four of them ELISA BP180–NC16A-positive. The three BP180–NC16A-negative ELISAs were immunoblot-positive on amniotic membrane extract, detecting each with band(s) at 180, 120, or 180 and 120 kDa. These serological findings along with IEM observations showed that gliptin-associated MMP autoantibodies could target the NC16A epitope, the mid-portion and the C-terminal domain of BP180. IEM of patient 8’s biopsy showed deposits exclusively at the upper LL, his BP180–NC16A ELISA was negative and immunoblotting detected a 200-kDa band consistent with the β4 chain of α6β4 integrin. Laminin 332 MMP, MM-EBA, and MM-LABD were excluded for all gliptin-associated MMPs.

Using three accountability methods, WHO–UMC’s criteria, Naranjo’s score (most used worldwide), and Begaud’s method (most used in France and Europe), we assessed gliptin imputability in MMP induction. With the WHO–UMC accountability criteria, gliptin triggering of MMP was **probable** for 17 patients and **unlikely** for 7. With Naranjo’s system, ADRs were considered **probable for** only 1 patient, **possible** for 16, and **doubtful** for 7 patients. Last, according to Begaud’s method, with scores ranging from I0 to I6, only 5 patients (I4 for three, I3 for two) were given **high accountability** scores, 12 had low **accountability** (I2 for eight, I1 for four), and 7 were scored I0, meaning chronologically **incompatible** or **undetermined** challenge.

The results of this study demonstrated that gliptins are probably responsible for some MMPs. Hence, all doctors prescribing gliptins must be made aware of this potential toxicity. The practical consequence of that finding is that, as soon as a positive accountability score is established, by precaution, gliptins, which can be easily switched in the case of inefficacy or ADR, should be replaced by another antidiabetic drug. Importantly, all such cases must be reported as possible ADRs to a pharmacovigilance center.

Large case–non-case comparative studies need to be performed to confirm or refute MMP induction by gliptins and better understand their pathogenic mechanism. Target-epitope mapping might help to determine whether a particular immune response occurs in drug-induced MMP.

## Ethics Statement

This study was approved by our local Institutional Review Board (IRB 00003835 no. 2013/39NI). All subjects gave written informed consent in accordance with the Declaration of Helsinki.

## Author Contributions

OG, VS, FC, and CP-S conceived and designed the study. MA, GB, SI-H-O, CB, PS, and CP-S collected clinical data. FA and SM-G conducted the immunological studies. OG, VS, BM, and CP-S organized the database. VS and OG conducted the statistical analyses. OG wrote the first draft of the manuscript. OG and CP-S rewrote sections of the manuscript. All authors contributed to manuscript revision, and read and approved the submitted version.

## Conflict of Interest Statement

The authors declare that the research was conducted in the absence of any commercial or financial relationships that could be construed as a potential conflict of interest.

## Abbreviations

BMZ, basement membrane zone; BP, bullous pemphigoid; DIF, direct immunofluorescence; DPPIV, dipeptidyl peptidase-IV; EBA, epidermolysis bullosa acquisita; ELISA, enzyme-linked immunosorbent assay; IEM, immunoelectron microscopy; IIF, indirect immunofluorescence; LABD, linear IgA bullous dermatosis; MMP, mucous membrane pemphigoid; SSS, salt-split skin; WHO–UMC, World Health Organization–Uppsala Monitoring Center.
