# Supplementary material for: Gliptin Accountability in Mucous Membrane Pemphigoid Induction in 24 Out of 313 Patients
Source: Front Immunol. 2018 May 24;9:1030. doi: 10.3389/fimmu.2018.01030 (PMC5976795; doi:10.3389/fimmu.2018.01030)
Supplement: Supplementary file 1 [file table_1.PDF]

Supplemental Table S1. Case reports of gliptin-induced bullous pemphigoid (BP).

1st author, Year (ref n°)	Patient sex/age, yr	Gliptin	Weeks to BP onset	Treatment	Dechallenge/ time to it	Outcome	Outcome after dechallenge	Paper's accountability score
Pasmatzi, 2011 (1)	F/59	Vildagliptin	8	Systemic CS	Yes/immediately	CR, 10 wk	Suggestive	None
	M/67	Vildagliptin	8	Doxycycline	Yes/immediately	CR, 8 wk	Suggestive	None
Skandalis, 2012 (2)	F/78	Vildagliptin	52	Systemic CS + MTX	Yes/NR	PR, 2 mo	Suggestive	WHO-UMC
	F/80	Sitagliptin	16	Systemic CS/MTX	Yes/NR	CR, 6 wk	Suggestive	WHO-UMC
	F/72	Vildagliptin	32	Topical CS	Yes/immediately	CR, 6 wk	Suggestive	WHO-UMC
	M/67	Vildagliptin	40	Systemic CS	Yes/immediately	CR, 5 d	Suggestive	WHO-UMC
	M/75	Vildagliptin	8	Systemic CS/MTX	Yes/immediately	CR	Suggestive	WHO-UMC
Aouidad, 2013 (3)	M/61	Vildagliptin	24	Topical CS	Yes/immediately	Clinical control, 1 wk; CR, 2 mo	Suggestive	None
	M/93	Sitagliptin	24	Topical CS	Yes/10 mo	Relapse before dechallenge; PR, 2 wk thereafter	Suggestive	None
	M/76	Sitagliptin	20	Topical CS	Yes/6 mo	Clinical control	Suggestive	None
Béné, 2014 (4)	F/86	Vildagliptin	4	Systemic CS	Yes/NR	Clinical control	Suggestive	None
	M/79	Vildagliptin	148	Clobetasol	Yes/3 mo	Relapse before dechallenge; Clinical control thereafter	Suggestive	None
	F/77	Vildagliptin	104	Topical CS	Yes/NR	Clinically improved	Suggestive	None
Attaway, 2014 (5)	M/70	Sitagliptin	48	Systemic CS	Yes/4 d	Clinical control, 3 d; CR, 3 mo	Suggestive	Naranjo's
Garcia, 2016 (6)	F/74	Vildagliptin	48	Systemic CS	Yes/15 mo	Clinical control	Suggestive	Karch–Lasagna
Mendonça, 2016 (7)	M/82	Linagliptin	6	Systemic CS	Yes/3 wk	Relapse before dechallenge	Suggestive	WHO-UMC
	F/77	Vildagliptin	NR	Systemic CS	Yes/NR	Lost-to-follow-up	NR	WHO-UMC
	F/72	Vildagliptin, then sitagliptin	12	Systemic CS	Yes/10 mo	Clinical control after dechallenge	Suggestive	WHO-UMC
Haber, 2016 (8)	M/60	Linagliptin	16	Topical CS	Yes/2 wk	CR, 1 wk; no relapse	Suggestive	None
	F/70	Linagliptin	4	Topical CS	Yes/immediately	CR, 5 d; no relapse	Suggestive	None
Sakai, 2017 (9)	F/76	Linagliptin	36	Topical CS	Yes/NR	Clinical control, 1 wk; CR, 16 wk	Suggestive	None
Keseroglu, 2017 (10)	F/61	Vildagliptin	48	Topical CS	Yes/immediately	CR, 3 wk	Suggestive	None
Fania, 2017 (11)	M/62	Linagliptin	24	NR	Yes/NR	NR	NR	Naranjo's
	M/77	Linagliptin	4	NR	No	NR	NR	Naranjo's
	F/73	Sitagliptin	32	NR	No	NR	NR	Naranjo's
	M/82	Alogliptin	8	NR	No	NR	NR	Naranjo's
	F/63	Vildagliptin	4	NR	No	NR	NR	Naranjo's
Yoshiji, 2017 (12)	M/81	Linagliptin	36	Systemic CS	Yes/NR	CR, wk	Suggestive	None
	F/86	Linagliptin	36	Systemic CS	Yes/NR	CR, 4 wk	Suggestive	None
	F/83	Sitagliptin	100	IVIg	Yes/NR	CR, 2 wk	Suggestive	None
	F/86	Vildagliptin	24	Systemic CS	Yes/NR	CR, 4 wk	Suggestive	None
	M/63	Anagliptin	20	Systemic CS	Yes/NR	CR, 2 wk	Suggestive	None
Harada, 2017 (13)	M/78	Sitagliptin	144	Systemic CS	Yes/NR	Died 14 d after BP diagnosis	Died	None
Schaffer, 2017 (14)	M/70	Vildagliptin Saxagliptin Sitagliptin	48	MTX	No	Relapse	NR	WHO-UMC
	F/84	Sitagliptin	144	Doxycycline	Yes/NR	CR, 8-mo follow-up	NR	WHO-UMC

M/73	Vildagliptin	>192	Topical CS	Yes/NR	CR, 52-mo follow-up	NR	WHO-UMC
F/70	Vildagliptin	20	Topical CS	No	3 relapses, 45-mo follow-up	NR	WHO-UMC
M/68	Vildagliptin Sitagliptin	120	Topical CS + MTX	Yes/NR	CR, 32-mo follow-up	NR	WHO-UMC
M/80	Sitagliptin	192	Topical CS	Yes/NR	CR, 63-mo follow-up	NR	WHO-UMC
M/85	Vildagliptin	>192	Topical CS	No	CR, 68-mo follow-up	NR	WHO-UMC
M/93	Vildagliptin	84	Topical CS	No	CR, 72-mo follow-up	NR	WHO-UMC
M/76	Sitagliptin	40	Topical CS	No	CR, 1 relapse	NR	WHO-UMC

F, female; M, male; wk, week(s); mo, month(s); d, day(s); CS, corticosteroids; CR, complete remission; MTX, methotrexate; NR, not reported; PR, partial remission.

[a] World Health Organization–Uppsala Monitoring Center system applied by Mendoça et al. in their review of the literature.

[b] Naranjo's scores were calculated by this paper's authors from the available reported information. The criteria used by the original papers' authors to differentiate possible from probable adverse drug reactions were rarely explained. More specifically, gliptin-induced BP most often had classical characteristics. Only non-inflammatory BP seem to be linked to gliptin intake. Without clinical specificity, those BPs could have been idiopathic, and therefore should have been classified as possible adverse drug reactions.

[c] BP onset >92 weeks after starting gliptin.
