# Supplementary material for: Gliptin Accountability in Mucous Membrane Pemphigoid Induction in 24 Out of 313 Patients
Source: Front Immunol. 2018 May 24;9:1030. doi: 10.3389/fimmu.2018.01030 (PMC5976795; doi:10.3389/fimmu.2018.01030)
Supplement: Supplementary file 2 [file table_2.PDF]

Supplemental Table S2. Characteristics of patients with gliptin-associated autoimmune bullous diseases (AIBDs).

**Characteristic**	**Case reports (1-14) *n* = 40**	**Béné et al. (15) *n* = 42**	**Benzaquen et al. (16) *n* = 28**	**This study *n* = 17**
Gliptin-associated, *n*	42 BPs	42 (28%) BPs among 150 ADRs[a]	28 (46%) BPs among 61 diabetics[b]	24 (38%) MMPs among 64 diabetics
Age, mean (range), years	75 (59–93)	74 (45-91)	79.1 +/- 7	69 (48–81)
Female/male, *n*, sex ratio	19/21 (0.9)	18/24 (0.75)	11/17 (0.65)	9/8 (1.1)
Time to AIBD onset, median (range)	32 (4–192) wk	40 wk (8 d–148 wk)	33 wk (10 d–156 wk)	136 (4–588) wk
Vildagliptin, *n* (%)	19 (45%)	31 (74%)	14 (50%)	11 (65%)
Sitagliptin, *n* (%)	10 (24%°	10 (24%)	10 (36%)	5 (29%)
Saxagliptin, *n* (%)	8 (19%)	1 (2%)	4 (14%)	1 (6%)
Other gliptin(s), *n* (%)	5 (12%)[c]	No	No	No
**Dechallenge** (suggestive), *n*	33 (26)	37 (34)	19 (18)	11 (7)
Time to AIBD clinical control, median (range)	NR	10 (5–15) d	Usual	8 (2–16) wk
**Rechallenge**, *n*	0	1 (positive)	1 (positive)	1 (positive)
**No dechallenge** (suggestive), *n*	8 (3)	5 (nd)	9 (4)	6 (2)

BP, bullous pemphigoid; ADRs, adverse drug reactions; MMP, mucous membrane pemphigoid; wk, week(s); d, day(s); NR, not reported.

[a] Compared vs. 0.6% of non-cases (odds ratio 67.5; 95% CI 47.1–96.9).

[b] Compared vs. 18% of non-cases (odds ratio 2.64; 95% CI 1.19–5.85).

[c] Other gliptins: 3 mixed, 1 alogliptin, 1 anagliptin.
